# Efficacy of deep brain stimulation in treating monogenic dystonia symptoms: protocol for a systematic review

**DOI:** 10.1136/bmjopen-2023-083127

**Published:** 2025-04-09

**Authors:** Beatriz Carmona-Hidalgo, Javier Quintero, Rocío Rodríguez-López, Juan Antonio Blasco-Amaro, Sylvia Boesch, Carola Reinhard

**Affiliations:** 1 Health Technology Assessment Area (AETSA), Andalusian Public Foundation Progress and Health (FPS), Seville, Spain; 2 Center for Rare Movement Disorders, Department of Neurology and Neurosurgery, Medical University Innsbruck, Innsbruck, Austria; 3 Institute for Medical Genetics and Applied Genomics, University of Tübingen, Tübingen, Germany; 4 Centre for Rare Diseases, University Hospital Tübingen, Tübingen, Germany

**Keywords:** Neurology, NEUROPATHOLOGY, Clinical Protocols, Systematic Review, NEUROSURGERY

## Abstract

**Abstract:**

**Introduction:**

Complexity leads to some dystonias being considered as rare diseases with scarce synthesised evidence. Despite the deficit of scientific evidence, deep brain stimulation (DBS) is currently an effective treatment for dystonias using different brain targets, providing significant improvement of dystonic symptoms regardless of their cause. However, there is considerable variability and non-response rate due to factors such as classification, semiology, duration, aetiology and genetic cause of the disease. This protocol presents the methodology of a planned systematic review to assess the efficacy of DBS as a treatment for monogenic dystonia symptoms, a broad spectrum of pathogenic dystonias due to variants in single genes not yet explored.

**Methods and analysis:**

This protocol follows the Preferred Reporting Items for Systematic review and Meta-Analysis Protocols guidelines. With the aim to test the efficacy of DBS in monogenic dystonias, the research question in population, intervention, comparator and outcomes format will cover patients with monogenic dystonia treated with DBS with a minimum of 3 months' follow-up after surgery. The outcomes will be assessed using generic and specific scales to measure the efficacy and safety of the intervention. The search will be performed in generic and specific databases and bibliographic resources from 2000. We will include systematic reviews, randomised controlled trials and primary studies in English. In this protocol, the initial search strategy in MEDLINE is presented. Additionally, the protocol provides a description of the prospective assessment of the risk of bias in the selected studies. If studies appear homogeneous and the sample of patients is sufficiently large, a meta-analysis and a subgroup analysis are planned.

**Ethics and dissemination:**

Ethics committee approval is not required. The results of the review will be published through an open access journal.

**PROSPERO registration number:**

**CRD42023448145:**

## Introduction

 Dystonia, defined as a condition characterised by sustained or intermittent muscle contractions causing abnormal, often repetitive, movements, postures or both, is a common movement disorder leading to generalised, segmental or focal impairment of motor activities and physiological postures.^1^ Because of the wide clinical spectrum and the association with other movement disorders, dystonias are often underdiagnosed or misdiagnosed in clinical practice. Therefore, genetics is an essential tool for classification which could allow a better understanding of the pathophysiology of the disease.^2^ There is a spectrum of genetic variants that converge towards the alteration of neuronal connections, causing a wide range of different phenotypes of dystonic alterations. It has been linked to over 200 genetic variants leading to autosomal dominant, recessive and X-linked disorders. To help clinicians reach the appropriate diagnosis and treatment procedures, several classifications have been proposed over time. The first classifications and definitions of dystonia were presented in the ’80s.^3 4^ The definition has evolved over time from primary dystonia (hereditary pattern) to secondary dystonia (hereditary neurological conditions or environmental causes) and psychological forms of dystonia. Later, in 2013,^5^ a consensus opinion proposed a new classification in two axes (clinical characteristics, aetiology) to incorporate the pathology, inheritance, age at onset and body distribution. Another scheme used for organising inherited dystonias is based on the DYTn coding system established by the Human Genome Organisation Gene Nomenclature Committee.^6^ This system assigns labels to gene loci defined by linkage analyses, and it is used to classify inherited dystonias. Monogenic dystonias were designated with a standard ‘DYT’ prefix and extend from DYT1 to DYT29. Of those with confirmed monogenic cause, DYT1-6, 11, 12 and 25–29 can present in childhood or early adulthood.^7^ In 2016, the Movement Disorder Society Task Force presented a new system for the Nomenclature of Genetic Movement Disorders with the aim of improving the previous naming system of the list of genes causing monogenic movement disorders. This system has been recently updated in 2022.^8^

Genetic dystonias may present with variable age at onset, body distribution, temporal pattern and associated features. In most cases, the cause is unknown. Dystonias are normally long-term diseases that require long-term treatment.^9^ The medical therapeutic treatments used in practice are quite extensive, but for many of these interventions, formal proof of efficacy is lacking.^10^ Deep brain stimulation (DBS) of the globus pallidus internus (GPi) is used as the main treatment of various forms of dystonia, but other brain targets are also relevant, such as subthalamic nucleus (STN), ventrointermediate nucleus (VIM) or pedunculopontine nucleus. DBS involves a surgical procedure to place electrical stimulators in the brain, connected to a battery, which deliver electrical impulses to the brain over time.^11^ DBS is usually considered to be an effective and safe therapeutic option for severe cases only, once other treatments have failed.^9 12^ It is a highly effective therapy for primary generalised and focal dystonias, but therapeutic success is compromised by a non-responder rate of up to 20%, which highlights the importance of patient selection.^13^ It is crucial to differentiate between monogenic dystonias and dystonias due to other causes to make a proper diagnosis and to determine if DBS is the best management option.^7^ The concept of routine genetic testing of patients prior to DBS intervention may help guide expectations for patients and clinical experts.^14 15^

To date, there are several published reviews assessing the use of DBS for different types of dystonias, confirming the beneficial effects of DBS on improving motor disabilities and the use of GPi as the main brain target.^19 16^,^18^ Since there are no large-scale studies with consistent evidence addressing DBS in dystonias caused by single gene variants and since the outcomes vary greatly across patients, this review aims to identify the current knowledge on the use of DBS as the best treatment strategy for monogenic dystonias. Different brain targets, genes and patient backgrounds will be explored to help achieve a better understanding and clinical management of these still uncertain types of dystonias. For that purpose, the main outcome of interest will be to measure the efficacy of DBS by assessing the clinical improvement of patients and quality of life through rating scales, as well as the adverse effects derived from the treatment.

## Methods and analysis

This protocol follows the Preferred Reporting Items for Systematic review and Meta-Analysis (PRISMA) Protocols guidelines^19^ (online supplemental data 1). The final systematic review will be conducted according to the PRISMA statement.^20^

### Eligibility criteria

The objective of this systematic review is to answer the following research question in population, intervention, comparator and outcomes format: *what is the efficacy of DBS in treating monogenic dystonia symptoms?,* presented in table 1. Articles will be eligible for inclusion if they are primary studies (case reports, case series and cohort studies), systematic reviews and randomised control trials, excluding narrative reviews, conference abstracts, editorials, letters to the editor and clinical practice guidelines. Studies with incomplete data and those whose full text is unavailable will be excluded. Articles must include patients with monogenic dystonia with genetic confirmation of the disease. Patients of all ages will be included. Since it is a rare disease, comparators will not be used. DBS will be used as an intervention using different brain targets. During our search, we will be monitoring the efficacy of DBS in treating monogenic dystonia measured by rating scales, quality of life indicators or patient-reported outcome measures (PROMS) and adverse events as measures of safety during the follow-up of the patient. Only studies with a minimum of 3 months of follow-up after DBS intervention will be included. No maximum follow-up time will be established. This protocol has been previously registered in the International Prospective Register of Systematic Reviews (PROSPERO) repository with the identification CRD42023448145.

**Table 1:** Elements of the clinical question translated into PICO format along with the inclusion and exclusion criteria to assess the efficacy of DBS in treating monogenic dystonia symptoms

What is the efficacy of deep brain stimulation in treating monogenic dystonia symptoms?		
Criteria	Inclusion criteria	Exclusion criteria
Population	Patients from all ages with monogenic dystonia who have a genetic confirmation of the disease. Genes causing monogenic dystonia: TOR1A, TAF1, SGCE, GNAL, KMT2B, PANK2, GNA01, GNB1, VPS16, THAP1, ATP1A3, ANO3, ADCY5, TUBB4A, PRKRA, GCH1, TH, EIF2AK2, VPS41, HPCA, AOPEP.	Non-human studies. Studies with assumed but not confirmed genetic data.
Intervention	DBS of the globus pallidus internus (GPi). DBS of the subthalamic nucleus (STN). DBS of the ventrointermediate nucleus (VIM). DBS of the pedunculopontine nucleus (PPN). Other targets.	Interventions not related to monogenic dystonia.
Comparator	None	None
Outcomes	Clinical improvements reported by clinicians and patients assessed by specific or generic rating scales for dystonia. Efficacy of DBS Safety of DBS Adverse events of DBS Quality of life or activities of daily living (ADL) or PROMS Medication regimen changes	Outcomes not related to monogenic dystonia.
Study design	Primary studies (case reports, case series, cohorts) Systematic reviews Randomised control trials	Clinical Practice Guidelines Narrative reviews Conference abstracts Editorials Letters to the editor

DBS, deep brain stimulation; PROMS, patient-reported outcome measures.

### Search strategy

The search will be carried out in the following core and subject-specific databases: MEDLINE (Ovid), Embase (Elsevier), the Cochrane library (Wiley), ORPHANET, EURORDIS, NORD, RARE-Bestpractices and GeneReviews. Other bibliographic resources will be PsycINFO (EBSCO), CINAHL (EBSCO), the Web of Science Core Collection and the INAHTA database. The searches will include both controlled language (descriptors) and free text.^21^ The preliminary search, developed in MEDLINE (Ovid), is available in online supplemental data 2. This search will be adapted to the rest of the databases. The search terms are related to dystonia as a disease, the dystonic disorders and the identified associated genes (*eg, TOR1A*, *KMT2B*) or genetic nomenclature (eg, *DYT1*). Regarding the intervention, the terms are DBS, intracranial or intracerebral stimulation. The searches will be restricted to the literature published in English and the date limit will be from 2000 onwards, since the use of DBS for dystonia was approved by the Food and Drug Administration in 2003. Similarly, DBS was marked in Europe and approved by the European Medicines Agency as CE (*Conformité Européenne*) (17).

### Screening and article selection

**Figure 1:**
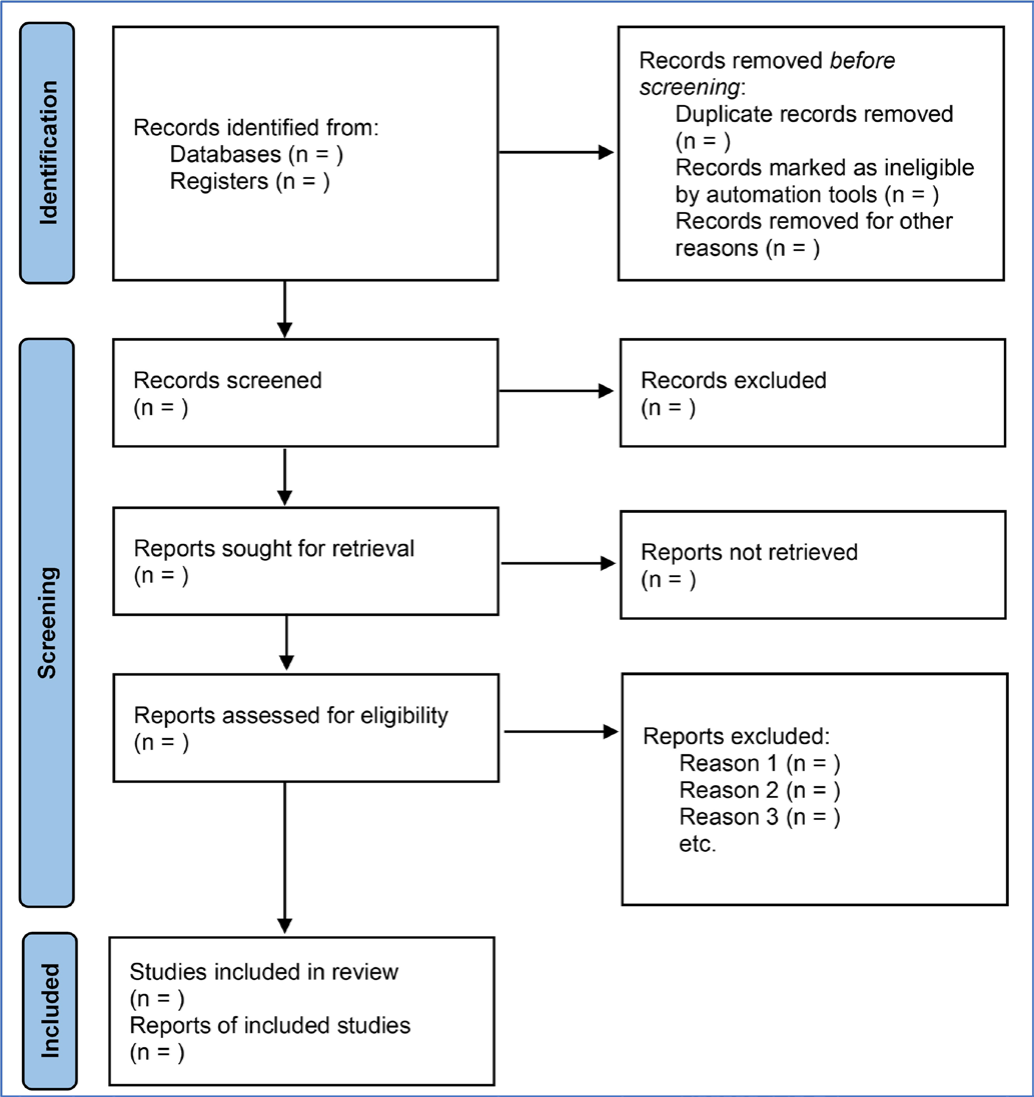
Information flow through the different phases of the selection process of a systematic review following Preferred Reporting Items for Systematic review and Meta-Analysis guidelines.

The screening process will be carried out through different phases following the PRISMA guidelines.^20^ The retrieved records from the literature search will be imported to Covidence, a systematic review data management platform (https://www.covidence.org), and the duplicates will be removed. First, two reviewers will independently screen the titles and abstracts, and after that, the full text (figure 1). Any disagreements will be resolved by discussion to achieve a consensus. If it is not achieved, a third reviewer will be consulted.

### Data extraction

The full text of each included article in the final set will be assessed by two independent reviewers to extract the data. Any discrepancies between reviewers will be discussed and resolved by an additional reviewer if a consensus cannot be achieved. The data extraction will be done using Excel spreadsheets. The data to be extracted will be specific details of the studies (author, year of publication, study type and location), the patients (age, gender, genetic variant, genetic test, comorbidities and follow-up time) and intervention (medication, brain target, age at onset, rating scales and clinical improvement, among others). An a priori template is presented in table 2. It is expected to extract individual patient data. If necessary, study authors will be contacted to request any necessary missing information.

**Table 2:** Initial planning of data to be extracted from the selected studies related to the efficacy of DBS in treating monogenic dystonia symptoms

Article data	Article type
	Title
	Year
	Authors
	Objective
	Country
Patient data	Number of patients
	Gender
	Current age
	Country
	Age at dystonia diagnosis
	Genetic tests
	Dystonia gene
	Inheritance
	Follow-up time
	Other diseases
Pharmacological treatments (before/after DBS)	Drug
	Dose
	Efficacy
Intervention	DBS target
	Age at DBS
	% improvement
Patient evaluation before DBS	Rating scales
	Value
Patient evaluation after DBS	Rating scales
	Value
	% improvement
Other data	quality of life, adverse events.

DBS, deep brain stimulation.

### Outcomes

We will consider different rating scales as outcome measures. There are the main ones among others:

- The Burke‐Fahn‐Marsden Dystonia Rating Scale (BFMDRS) is an instrument for the quantitative assessment of dystonia in both children and adults.^22^
- The Toronto Western Spasmodic Torticollis Rating Scale is used to assess the severity of cervical dystonia (assessing severity, disability and pain) and the success of treatment.^23^
- The Unified Dystonia Rating Scale is designed to assess the severity of dystonia in individual body areas. Each body region is^24^ rated for dystonia severity and duration.
- Visual analogue scales are psychometric measuring instruments designed to document the characteristics of disease-related symptom severity in individual patients to achieve a rapid (statistically measurable and reproducible) classification of symptom severity and disease control.^25^
- The Clinical Global Impressions Severity asks the clinician one question: ‘considering your total clinical experience with this particular population, how ill is the patient at this time?’, which is rated on a seven-point scale.^26^
- The Brief Psychiatric Rating Scale measures psychiatric symptoms such as depression, anxiety, hallucinations and unusual behaviour. It evaluates 24 items with scores from 1 to 7.^27^
- The Body Image Disturbance Questionnaire is used for the assessment of body image disturbance. It is a 7-item questionnaire.^28^

**STRENGTHS AND LIMITATIONS OF THIS STUDY**

- This protocol presents the methodology of the first systematic review on deep brain stimulation as a treatment for monogenic dystonias.
- The protocol adheres to the Preferred Reporting Items for Systematic Reviews and Meta-Analyses for Protocols guidelines.
- A rigorous methodological process will be followed to answer the research question: *what is the efficacy of deep brain stimulation to treat monogenic dystonia symptoms?*
- The outcomes will be assessed using all applied dystonia scales to measure the clinical improvements of the patients after the surgery.
- A meta-analysis along with subgroup analysis is planned to identify the efficacy and safety of the intervention.

Quality of life and PROMS, efficacy (symptom improvement after surgery, period of stimulation, number and type of stimulated brain targets, relapse time, dose-response effect and tolerance), safety and adverse events (serious or non-serious adverse events, infections derived from the device implantation and device failures) of DBS and medication regimen changes will also be retrieved. It could be possible to find new rating scales, surveys or other ways to assess the outcomes of the intervention during data analysis.

Patients with >25% improvement in the BFMDRS will be considered as DBS responders.^29^ Moreover, we will assess DBS response according to the minimal clinically important change defined for each dystonia scale, whenever available.

### Risk of bias and quality of articles

The quality of every single selected article will be assessed using different evaluation tools depending on the type of article. For systematic reviews, risk of bias will be assessed using the AMSTAR-II tool,^30^ randomised controlled trials by Cochrane Collaboration Risk of Bias 2 tool,^31 32^ QUADAS tool will be used for diagnostic accuracy studies,^33^ the Newcastle-Ottawa Scale^34^ will be used for cohort studies, and the tool developed by Murad *et al*^35^ for case reports and case series.

The assessment will be performed by two authors independently, and the disagreements will be resolved by discussion. If an agreement is not achieved, a third reviewer will be invited. The risk of bias assessment results will be summarised in tables and text in the completed review.

### Data synthesis

Information about patients and study characteristics will be collected and presented in a tabular form. If there are enough studies to quantitatively synthesise the results, heterogeneity between studies will be calculated using the I^²^ statistic. Random effects meta-analysis will be conducted with 95% CIs. If possible, pooled estimates of OR/RR with 95% CIs will be calculated, and a sequential analysis to yield an accurate effect size on the efficacy of the intervention will be carried out. If the sample of patients is sufficiently large, this information could be separated into subgroups, such as age of the patients (children, <18 years, and adults), gender, gene variants, brain targets, rating scales, study types, follow-up time or other relevant factors and outcomes. The analyses will be conducted with RevMan (https://training.cochrane.org/online-learning/core-software/revman), a systematic review and meta-analysis tool designed by Cochrane. However, this might not be feasible if studies are too heterogeneous or there is a lack of reported data.^36^ In this case, a narrative synthesis of the findings will be performed.

## Discussion

This review aims to systematically address for the first time the evidence of the use of DBS for monogenic dystonia. DBS represents an important treatment option for dystonias when generalised or once previous pharmacological treatments have failed. Different target nuclei for DBS have been studied in people with dystonia, including the GPi, the STN and the VIM.^9 11^ In this light, there is also increasing interest in DBS technique for the treatment of monogenic dystonia, and several reviews have been published up to now.^19 16^,^18^ However, the evidence on the best treatment strategies for monogenic dystonia is still uncertain, thus posing a major obstacle to stratifying and advising patients on a potentially highly effective treatment.

DBS GPi for dystonias was introduced in 2003 in a cohort of patients with generalised or segmental dystonias, especially in carriers of DYT1 mutations, in whom treatment options have been limited. ‘Golden responders’ to DBS GPi were mostly patients carrying a DYT1 mutation. Conversely, short-term and long-term DBS effects in carriers of mutations of recently discovered dystonia genes who underwent GPi DBS for generalised or segmental dystonia in young age are seemingly highly variable. Furthermore, the value of preinterventional genetic testing is currently unclear, and DBS target structures for genetically assigned monogenic dystonias are to be studied. While the optimal time point of intervention for DBS in dystonias is still under debate, cumulative evidence suggests that an early intervention may decisively contribute to a successful DBS outcome,^1 37 38^ raising the issue of a timely genetic diagnosis.

We anticipate that the extent of available evidence in the literature may be a main limitation to this study. There could be limitations if the follow-up time after surgery is not enough to measure clinical improvements in the patients. The topic focuses on monogenic dystonias caused by gene variants reported in the literature to date, although it must not be ruled out that there are other variants that have not been reported or that share features with other more complex dystonias. Heterogeneity can be anticipated, in terms of types of studies, background of the patients and the quantity and quality of data reported. Considering that the symptoms resulting from dystonia may vary among patients and in the way they are reported by the studies, variability in the analysis could make it easier to reach conclusions about certain symptoms than others. The evaluation of patient-relevant hard outcomes such as hospitalisations, all-cause mortality and serious adverse events would be of utmost relevance, but it is unfortunately poorly addressed in monogenic dystonias.^39^ In addition, it could be difficult to compare the effects, including quantitative and qualitative data. To address these limitations, a division of patients into subgroups of similar characteristics could be performed. Articles could also be grouped by type of study (systematic reviews, randomised controlled trials and primary studies) or by the rating scales used to report the outcomes. Studies of qualitative outcomes will be separated from quantitative ones.

We anticipate that the findings from this review will provide insight into the efficacy and safety of DBS in treating monogenic dystonia symptoms. This will open new horizons for the clinical research of this pathology in the future, and it will establish the bases of specific or deeper studies for populations with monogenic dystonias. The results will be disseminated through publication due to the potential to impact on the clinical management of this disease.

## Ethics and dissemination

Ethics committee approval is not required. The final results of this review will be submitted for publication in a peer-reviewed open-access journal. Any deviations from this protocol will be recorded and explained in the final report.

## Supplementary material

10.1136/bmjopen-2023-083127 online supplemental file 1

10.1136/bmjopen-2023-083127 online supplemental file 2
