# Supplementary material for: Efficacy of deep brain stimulation in treating monogenic dystonia symptoms: protocol for a systematic review
Source: BMJ Open. 2025 Apr 9;15(4):e083127. doi: 10.1136/bmjopen-2023-083127 (PMC11987142; doi:10.1136/bmjopen-2023-083127)
Supplement: online supplemental file 1 [file bmjopen-15-4-s001.pdf]

Supplementary Data 1. Reporting checklist for protocol of a systematic review and meta-analysis based on PRISMA-P guidelines.

		Reporting Item	Page Number
**Title**			
**Identification**	#1a	Identify the report as a protocol of a systematic review	Mentioned in *title section.*
**Update**	#1b	If the protocol is for an update of a previous systematic review, identify as such	This protocol is not an update of a previous review.
**Registration**			
	#2	If registered, provide the name of the registry (such as PROSPERO) and registration number	PROSPERO (CRD42023448145).
**Authors**			
**Contact**	#3a	Provide name, institutional affiliation, e-mail address of all protocol authors; provide physical mailing address of corresponding author	Mentioned in *authors and affiliations* sections.<br><br>Corresponding author:<br><br>Beatriz Carmona-Hidalgo<br>beatriz.carmona.hidalgo@juntadeandalucia.es
**Contribution**	#3b	Describe contributions of protocol authors and identify the guarantor of the review	Mentioned in *authors´ contributions* section.
**Amendments**			
	#4	If the protocol represents an amendment of a previously completed or published protocol, identify as such and list changes; otherwise, state plan for documenting important protocol amendments	It is a study protocol.
**Support**			
**Sources**	#5a	Indicate sources of financial or other support for the review	Mentioned in *funding statement* section.
**Sponsor**	#5b	Provide name for the review funder and / or sponsor	Not applicable.
**Role of sponsor or funder**	#5c	Describe roles of funder(s), sponsor(s), and / or institution(s), if any, in developing the protocol	Not applicable.
**Introduction**			
**Rationale**	#6	Describe the rationale for the review in the context of what is already known	Mentioned in *introduction* section.
**Objectives**	#7	Provide an explicit statement of the question(s) the review will address with reference to participants, interventions, comparators, and outcomes (PICO)	Mentioned in *introduction* section.
**Methods**			

**Eligibility criteria**	#8	Specify the study characteristics (such as PICO, study design, setting, time frame) and report characteristics (such as years considered, language, publication status) to be used as criteria for eligibility for the review	Mentioned in *eligibility criteria* (table 1) section.
**Information sources**	#9	Describe all intended information sources (such as electronic databases, contact with study authors, trial registers or other grey literature sources) with planned dates of coverage	Mentioned in *search strategy* section.
**Search strategy**	#10	Present draft of search strategy to be used for at least one electronic database, including planned limits, such that it could be repeated	Mentioned in *search strategy* section (Supplementary data 2).
**Study records - data management**	#11a	Describe the mechanism(s) that will be used to manage records and data throughout the review	Mentioned in *screening and article selection* section.
**Study records - selection process**	#11b	State the process that will be used for selecting studies (such as two independent reviewers) through each phase of the review (that is, screening, eligibility and inclusion in meta-analysis)	Mentioned in *screening and article selection* and *data synthesis* sections.
**Study records - data collection process**	#11c	Describe planned method of extracting data from reports (such as piloting forms, done independently, in duplicate), any processes for obtaining and confirming data from investigators	Mentioned in *data extraction* section.
**Data items**	#12	List and define all variables for which data will be sought (such as PICO items, funding sources), any pre-planned data assumptions and simplifications	Mentioned in *data extraction* section (table 2).
**Outcomes and prioritization**	#13	List and define all outcomes for which data will be sought, including prioritization of main and additional outcomes, with rationale	Mentioned in *outcomes* section.
**Risk of bias in individual studies**	#14	Describe anticipated methods for assessing risk of bias of individual studies, including whether this will be done at the outcome or study level, or both; state how this information will be used in data synthesis	Mentioned in *risk of bias and quality of articles* section.
**Data synthesis**	#15a	Describe criteria under which study data will be quantitatively synthesized	Mentioned in *data synthesis* section.
**Data synthesis**	#15b	If data are appropriate for quantitative synthesis, describe planned summary	Mentioned in *data synthesis* section.

		measures, methods of handling data and methods of combining data from studies, including any planned exploration of consistency (such as I2, Kendall's τ)	
**Data synthesis**	#15c	Describe any proposed additional analyses (such as sensitivity or subgroup analyses, meta-regression)	Mentioned in *data synthesis* section.
**Data synthesis**	#15d	If quantitative synthesis is not appropriate, describe the type of summary planned	Mentioned in *data synthesis* section.
**Meta-bias(es)**	#16	Specify any planned assessment of meta-bias(es) (such as publication bias across studies, selective reporting within studies)	Mentioned in *data synthesis* section.
**Confidence in cumulative evidence**	#17	Describe how the strength of the body of evidence will be assessed (such as GRADE)	Mentioned in *risk of bias and quality of articles* section.
