# Supplementary material for: Efficacy of deep brain stimulation in treating monogenic dystonia symptoms: protocol for a systematic review
Source: BMJ Open. 2025 Apr 9;15(4):e083127. doi: 10.1136/bmjopen-2023-083127 (PMC11987142; doi:10.1136/bmjopen-2023-083127)
Supplement: online supplemental file 2 [file bmjopen-15-4-s002.pdf]

Supplementary Data 2. Preliminary search strategy.

Research Question: *What is the efficacy of deep brain stimulation to treat monogenic dystonia symptoms?*

Database	Date	Search Name	Results
Base de datos: Ovid MEDLINE(R) and Epub Ahead of Print, In-Process, In-Data-Review & Other Non-Indexed Citations, Daily and Versions <1946 to July 11, 2023>	19/07/2023	MED RND Monogenic Dystonia DBS Final	529

1 Dystonia/ 7329

2 exp Dystonic Disorders/ 4594

3 Blepharospasm/ 1446

4 Torticollis/ 3963

5 (Dystoni* or Blepharospasm* or Torticollis*).ti,ab,kf. 24251

6 or/1-5 26777

7 (monogen* or (single adj5 (Mutat* or variant* or modificat* or alterat* or delet* or microdelet* or defect* or anomal* or abnormalit* or inherit* or heredit* or gene*))).ti,ab,kf. 149565

8 6 and 7306

9 (Dyston* and (TOR1A* or Torsina* or "Torsin 1A*" or TAF1* or SCGE* or GNAL* or KMT2B* or PANK2* or GNA01* or GNB1* or VPS16* or THAP1* or ATP1A3* or ANO3 or ADCY5* or TUBB4A* or PRKRA* or VPS41* or EIF2AK2* or HPCA* or AOPEP* or GCH1* or TH)).ti,ab,kf. 1334

10 (((Primary or Myoclon*) adj4 dystoni*) or "Segawa syndrome").ti,ab,kf. 1925

11 (DYT1* or DYT-1* or DYTTOR1A* or DYT-TOR1A* or DYT3* or DYT-3* or "DYT PARK-TAF1*" or DYTPARK* or DYT-SCGE* or DYTSCGE* or DYT11* or DYT-11* or DYT-GNAL* or DYTGNAL* or DYT25* or DYT-25* or DYT28* or DYT-28* or DYT-KMT2B* or DYTKMT2B* or DYT-PANK2* or DYTPANK2* or DYT-GNAO1* or DYTGNAO1* or DYT-GNB1* or DYTGNB1* or DYT30* or DYT-30* or DYT-THAP1* or DYTTHAP1* or DYT6* or DYT-6* or DYT-ATP1A3* or DYTATP1A3* or DYT12* or DYT-12* or DYT-ANO3* or DYTANO3* or DYT24* or DYT-24* or DYT-ADCY5* or DYTADCY5* or DYT4* or DYT-4* or DYT-TUBB4A* or DYTTUBB4A* or DYT-PRKRA* or DYTPRKRA* or DYT16* or DYT-16* or DYT-5* or DYT5* or DYT-GNAL* or DYTGNAL*).ti,ab,kf. 1042

12 or/8-113494
13 Deep Brain Stimulation/ 11151
14 ((brain or cerebral or Intracerebral* or Intracranial* or pallid* or subthalam* or Nucleus* or STN* or GPi* or VIM* or PPN*) adj5 stimulat*).ti,ab,kf. 41808
15 or/13-14 43186
16 and/12,15 565
17 limit 16 to (english language and yr="2000 - 2024") 529
