# Supplementary material for: Efficacy of deep brain stimulation in treating monogenic dystonia symptoms: protocol for a systematic review
Source: BMJ Open. 2025 Apr 9;15(4):e083127. doi: 10.1136/bmjopen-2023-083127 (PMC11987142; doi:10.1136/bmjopen-2023-083127)
Supplement: Supplementary file 3 [file bmjopen-2023-083127.reviewer_comments.pdf]

PEER REVIEW HISTORY

BMJ Open publishes all reviews undertaken for accepted manuscripts. Reviewers are asked to complete a checklist review form and are provided with free text boxes to elaborate on their assessment. These free text comments are reproduced below.

ARTICLE DETAILS

Title (Provisional)

Efficacy of deep brain stimulation to treat monogenic dystonia symptoms: protocol for a systematic review

Authors

Carmona-Hidalgo, Beatriz; Quintero, Javier; Rodríguez-López, Rocío; Blasco-Amaro, Juan Antonio; Boesch, Sylvia; Reinhard, Carola

---

VERSION 1 - REVIEW

**Reviewer**	**1**
**Name**	**Macerollo, A.**
**Affiliation**	**University of Liverpool**
**Date**	**13-Feb-2024**
**COI**	**None**

The paper is very well written.

My minor comment is to add a figure as flow chart to summarise the process of paper selection

---

**Reviewer**	**2**
**Name**	**Sarva, Harini**
**Affiliation**	**Weill Cornell Medicine, Department of Neurology**
**Date**	**03-Jun-2024**
**COI**	**First authored a systematic review and meta analysis of DBS for genetic dystonia that was published a few months ago**

Systematic review and meta analysis of this topic was just published a few months ago.

I'm not sure another complete review needs to be done. I would recommend a letter to the editor and have more recent papers from April 2023 onwards reviewed.

**Reviewer**	**3**
**Name**	**de Sá Ferreira, Arthur**
**Affiliation**	**Centro Universitario Augusto Motta, Postgraduate Program in Rehabilitation Sciences**
**Date**	**20-Sep-2024**
**COI**	**None to declare.**

Manuscript ID: bmjopen-2023-083127

Manuscript title: Efficacy of deep brain stimulation to treat monogenic dystonia symptoms: protocol for a systematic review

Comments

This manuscript reports a study protocol for a systematic review to assess the efficacy of deep brain stimulation as a treatment for monogenic dystonia symptoms. The topic is of interest to the field and the literature review suggests there is a gap for this study. The study protocol is prospectively registered (PROSPERO Registration Number: CRD42023448145). The reporting follows adequate guidelines (PRISMA-P) with the checklist made available. A comprehensive literature search is planned, and a full search strategy is provided. Statistical analysis plan is adequate for the study and anticipates important aspects such as subgroup analysis. Appraisal of the limitations is well reported. I have no comments for the authors to revise.

**Reviewer**	**4**
**Name**	**Løkkegaard, Annemette**
**Affiliation**	**Copenhagen University Hospital Bispebjerg, Department of Neurology**
**Date**	**17-Nov-2024**
**COI**	**Boston scientific, Course attendanceIpsen, Course attendance**

Overall, this is a relevant manuscript with an important clinical question.
It is nice to see a protocol for a systematic review, and the methodology described is thorough and up to date.

Comments:

1. In the manuscript, the authors state, that deep brain stimulation has been proven effective for dystonia (e.g., abstract: "*It has shown to provide significant improvement of dystonic symptoms regardless of it cause*" and introduction "*It is a highly effective therapy for primary generalized and focal dystonias*"). However,

the evidence behind this depends on the studies included. A Cochrane review from 2019 concluded that it may reduce symptoms but the evidence is very limited (1). It should be included what this statement was based on, or more nuances should be included.

2. In the description of background, the authors should refer to relevant literature:
   a. Dystonia is not a common disease, and the prevalence is low compared to other movement disorders such as Parkinson's disease, and essential tremor
   b. Dystonia classification should be mentioned already in the introduction (2). Here it is defined that the diagnosis of dystonia is based on two axes: *"clinical characteristics, including age at onset, body distribution, temporal pattern and associated features (additional movement disorders or neurological features); and etiology, which includes nervous system pathology and inheritance."* Genetic background is used in the search for etiology.
   c. The newest definitions regarding genetic nomenclature should be more precisely described, and the impact different nomenclatures could have in a comprehensive search of literature.

3. The authors include a thorough description of their systematic methods for searches, screening, article selection, and data extraction including a table of data to be extracted.
   Missing is:
   a. With the current search string for instance DYT/PARK-ATP1A3 would not be found. As the number of articles is limited it might be prudent to look for articles where monogenetic variants of any kind have been found.
   b. Follow up time is missing, used for data collection (e.g., data reported at the maximum follow up time?). Consider also including in the methods section, that observational studies will be included (as already described in Table 1).

4. In table 1 no comparators are mentioned. However, if the authors intend to include randomised clinical trials, comparators will be used (e.g., deep brain stimulation versus sham stimulation or best medical treatment).

5. The authors include several symptom severity scales as their primary focus. However, symptom severity scales have a lot of limitations (3). E.g., how should clinicians and patients interpret a change in score and what is the minimal important difference for these symptom scales? The authors should consider also including patient-relevant hard outcomes such as hospitalisations, all-cause mortality, and serious adverse events. If they do include symptom severity scales, a minimal important difference should be predefined in the protocol.

6. How come the authors use Cochranes risk of bias tool for randomised but not ROBINS-I tool for observational studies?

7. In the section on data synthesis, the authors mention a subgroup analysis on age. If any other subgroup analyses are planned, these should be predefined (even if these are not performed in the end due to insufficient data). Other

subgroup analyses of interest could be subtype of dystonia, study type (e.g., randomised clinical trials vs. observational studies), as mentioned in the discussion, or comparators for the randomised studies (e.g., sham stimulation vs. best medical treatment).

8. In table 1, the authors have included “Efficacy of DBS”, “Safety of DBS”, “Adverse events of DBS” as outcomes. How will data on these outcomes be extracted? This should be specified (e.g., serious adverse events or non-serious adverse events).

9. The authors do not employ any trial sequential analyses. Therefore, their results will be limited by the fact, that they will not be able to determine if their analyses are underpowered (e.g., to distinguish if there is no difference between deep brain stimulation and the comparator for randomised studies or if there is not enough data).

10. The discussion should be revised.
    Page 11, line 228: The sentence: “Parkinson's disease is the most common neurological disease for which DBS is used, providing a high rate of quality of life for patients (33). As a result, DBS represents an important treatment option for dystonias once previous pharmacological treatments have failed”. Should be revised. Although the connection may be relevant, it is not obvious why?

    The postulate that “There are three main issues that are unsolved in DBS for monogenic dystonias” is not adequately discussed. As these issues are not reflected in the background and the objective in the review, it is unclear why it is discussed.
    The sentence “Thus, short term and long-term DBS effects in recently discovered DYT mutation carriers with generalized or segmental dystonia in young age that underwent DBS GPi are highly variable” is unclear.
    The third issue: “The window of opportunity (optimal time point of intervention after genetic diagnosis) for DBS in dystonias is still under debate.” is a postulate and should be either dismissed or more thoroughly explained. In some forms of dystonia age might be important – is that what this refers to?

11. **Language**: Small errors (randomised or randomized, two or 2). The term “mutant genes” is no longer preferred, but gene variants.

1. Rodrigues FB, Duarte GS, Prescott D, Ferreira J, Costa J. Deep brain stimulation for dystonia. Cochrane Database Syst Rev. 2019;1:CD012405.
2. Albanese A, Bhatia K, Bressman SB, DeLong MR, Fahn S, Fung VS, et al. Phenomenology and classification of dystonia: a consensus update. Mov Disord. 2013;28(7):863-73.
3. Petersen JJ, Juul S, Kamp CB, Lokkegaard A, Jakobsen JC. We need to rethink outcomes in Parkinson's disease research. BMJ. 2024;387:q2280.

VERSION 1 - AUTHOR RESPONSE

Reviewer: 1
Dr. A. Macerollo, University of Liverpool
Comments to the Author:
The paper is very well written.
My minor comment is to add a figure as flow chart to summarise the process of paper selection.

Thank you for the comment. We have included a new figure (Fig.1) summarizing and explaining the screening process

Reviewer: 2
Dr. Harini Sarva, Weill Cornell Medicine
Comments to the Author:
Systematic review and meta analysis of this topic was just published a few months ago.
I'm not sure another complete review needs to be done. I would recommend a letter to the editor and have more recent papers from April 2023 onwards reviewed.

Thank you very much for the suggestion. In fact, as you have mentioned and is explained in the *introduction section* of the protocol, there are pre-existing studies in the use of DBS to treat dystonia symptoms. In the planned systematic review, we will focus on the study of monogenic dystonias, the wide range of genes causing the disease, the different brain targets using for DBS stimulation, and using all applied dystonia scales in order to retrieve all the available scientific evidence on this specific group of dystonias. Similarly, due to the heterogeneity in the response among the patients, it would be interesting to understand the responsible factors and to have the opportunity to study them from different patients' subgroups or backgrounds. On the other hand, in relation to the date indicated of April 2023, the bibliographic searches will be carried out in generic and specific databases from the year 2000 to the present (time of completion of the systematic review). This will allow updating the results to know the state of the art on monogenic dystonias and reduce the existing knowledge gap.

Reviewer: 3
Dr. Arthur Sá Ferreira, Augusto Motta University Centre
Comments to the Author:
Manuscript ID: bmjopen-2023-083127

Manuscript title: Efficacy of deep brain stimulation to treat monogenic dystonia symptoms: protocol for a systematic review

Comments

This manuscript reports a study protocol for a systematic review to assess the efficacy of deep brain stimulation as a treatment for monogenic dystonia symptoms. The topic is of interest to the field and the literature review suggests there is a gap for this study. The study protocol is prospectively registered (PROSPERO Registration Number: CRD42023448145). The reporting follows adequate guidelines (PRISMA-P) with the checklist made available. A comprehensive literature search is planned, and a full search strategy is provided. Statistical analysis plan is adequate for the study and

anticipates important aspects such as subgroup analysis. Appraisal of the limitations is well reported. I have no comments for the authors to revise.

Thank you for your comment.

Reviewer: 4

Dr. Annemette Løkkegaard, Copenhagen University Hospital Bispebjerg, University of Copenhagen
Comments to the Author:
Overall, this is a relevant manuscript with an important clinical question.
It is nice to see a protocol for a systematic review, and the methodology described is thorough and up to date.

Comments:
1. In the manuscript, the authors state, that deep brain stimulation has been proven effective for dystonia (e.g., abstract: “It has shown to provide significant improvement of dystonic symptoms regardless of it cause” and introduction “It is a highly effective therapy for primary generalized and focal dystonias”). However, the evidence behind this depends on the studies included. A Cochrane review from 2019 concluded that it may reduce symptoms but the evidence is very limited (1). It should be included what this statement was based on, or more nuances should be included.

Thank you for your observation. In fact, in the manuscript we have already mentioned the existence of previous systematic reviews in this field in which, despite the good results in motor disabilities obtained after the intervention, the variability among the patient's responses means that, the results are not totally consistent among the studies. Therefore, we have re-written part of these paragraphs to increase clarity on this point (lines 24-32, 109-119).

2. In the description of background, the authors should refer to relevant literature:
a. Dystonia is not a common disease, and the prevalence is low compared to other movement disorders such as Parkinson’s disease, and essential tremor

Thank you for the appreciation. We have included a recent reference related (ref 39).

b. Dystonia classification should be mentioned already in the introduction (2). Here it is defined that the diagnosis of dystonia is based on two axes: “clinical characteristics, including age at onset, body distribution, temporal pattern and associated features (additional movement disorders or neurological features); and etiology, which includes nervous system pathology and inheritance.” Genetic background is used in the search for etiology.
c. The newest definitions regarding genetic nomenclature should be more precisely described, and the impact different nomenclatures could have in a comprehensive search of literature.

Thank your both suggestions. In the introduction section we have re-written the paragraphs taking them into consideration to clarify these points (lines 72-90).

3. The authors include a thorough description of their systematic methods for searches, screening, article selection, and data extraction including a table of data to be extracted.
Missing is:
a. With the current search string for instance DYT/PARK-ATP1A3 would not be found. As the number

of articles is limited it might be prudent to look for articles where monogenetic variants of any kind have been found.

Thank you for your comment. We have designed a preliminary search strategy in the literature using controlled and free text to be as exhaustive as possible. As you have mentioned, the presence of gene variations would be reduced or not totally reported in the literature, so, we have planned to search in specific and general databases to cover all the possible scenarios. In the same way, the review of the grey literature and other complementary sources is planned.

b. Follow up time is missing, used for data collection (e.g., data reported at the maximum follow up time?). Consider also including in the methods section, that observational studies will be included (as already described in Table 1).

Thank you for the comment. We have defined a minimum follow-up time of 3 months after the intervention due to it is the minimum time to make an evaluation of DBS efficacy. However, a maximum follow-up time is not defined in order to analyse the responses of the patients to DBS in different times and evaluate possible differences (if any). Following your recommendations, we have added the comment in the *methods section* (lines 128-131, 138-139).

4. In table 1 no comparators are mentioned. However, if the authors intend to include randomised clinical trials, comparators will be used (e.g., deep brain stimulation versus sham stimulation or best medical treatment).

Thank you for your observation. Primarily, we have not included any comparator, as the available evidence in monogenic dystonias is limited and very heterogeneous, so we do not expect to find any defined reference method that can be used as a specific comparator or gold standard.

5. The authors include several symptom severity scales as their primary focus. However, symptom severity scales have a lot of limitations (3). E.g., how should clinicians and patients interpret a change in score and what is the minimal important difference for these symptom scales? The authors should consider also including patient-relevant hard outcomes such as hospitalisations, all-cause mortality, and serious adverse events. If they do include symptom severity scales, a minimal important difference should be predefined in the protocol.

We thank the reviewer for this thoughtful comment, which indeed addresses a key point, i.e. the heterogeneity of the assessment performed in DBS studies in dystonia. To i) take account of this heterogeneity and ii) avoid excluding large dataset thus reducing the strength of our conclusions, we decided to consider all the routinely used dystonia scales as endpoint. The findings of the review will be presented considering both i) the minimal clinically important change for each scale whenever available (1, 2) and ii) the empirically defined classification in responders versus non-responders, as applied in DBS trials in dystonia and in the routine (3). Similarly, another relevant outcome is the safety of DBS related to adverse events as you have mentioned and as already specified in *Outcomes section* and in *table 1*. The evaluation of outcomes such as hospitalisations, all-cause mortality, and serious adverse events would be of utmost relevance. Unfortunately, these outcomes are poorly or not at all address in monogenic dystonias. We commented on this point as further limitation in the *Discussion section* and cited here the literature suggested by the reviewer.

6. How come the authors use Cochranes risk of bias tool for randomised but not ROBINS-I tool for observational studies?

Thank you for your appreciation. Taking into consideration the use of ROBINS-I tool for the quality assessment of observational studies as you have mentioned, in this study and from previous works of the group, we have defined the use of the tool developed by Murad et al, 2018 (4) for the assessment of the quality of case reports and case series, and the Newcastle-Ottawa Scale (NOS) (5) for cohort studies. These tools are already widely validated and present the data in a comprehensive way.

7. In the section on data synthesis, the authors mention a subgroup analysis on age. If any other subgroup analyses are planned, these should be predefined (even if these are not performed in the end due to insufficient data). Other subgroup analyses of interest could be subtype of dystonia, study type (e.g., randomised clinical trials vs. observational studies), as mentioned in the discussion, or comparators for the randomised studies (e.g., sham stimulation vs. best medical treatment).

Thank you for the suggestion. We have included more details about subgroup analysis in *Data synthesis section* (line 234-237).

8. In table 1, the authors have included "Efficacy of DBS", "Safety of DBS", "Adverse events of DBS" as outcomes. How will data on these outcomes be extracted? This should be specified (e.g., serious adverse events or non-serious adverse events).

Thank you for the suggestion. We have included more details about outcomes in *Outcomes section* (lines 207-213).

9. The authors do not employ any trial sequential analyses. Therefore, their results will be limited by the fact, that they will not be able to determine if their analyses are underpowered (e.g., to distinguish if there is no difference between deep brain stimulation and the comparator for randomised studies or if there is not enough data).

Thank you for the suggestion. We have included this point in *Data synthesis section* (lines 232-234).

10. The discussion should be revised.
Page 11, line 228: The sentence: "Parkinson's disease is the most common neurological disease for which DBS is used, providing a high rate of quality of life for patients (33). As a result, DBS represents an important treatment option for dystonias once previous pharmacological treatments have failed". Should be revised. Although the connection may be relevant, it is not obvious why?

The postulate that "There are three main issues that are unsolved in DBS for monogenic dystonias" is not adequately discussed. As these issues are not reflected in the background and the objective in the review, it is unclear why it is discussed.

The sentence "Thus, short term and long-term DBS effects in recently discovered DYT mutation carriers with generalized or segmental dystonia in young age that underwent DBS GPi are highly variable" is unclear.

We agree with yours comments, so the sentences have been re-written to clarify these points in Discussion *section.*

The third issue: “The window of opportunity (optimal time point of intervention after genetic diagnosis) for DBS in dystonias is still under debate.” is a postulate and should be either dismissed or more thoroughly explained. In some forms of dystonia age might be important – is that what this refers to?
We apologize for the ambiguity. As per reviewer suggestion, we have reworded these passages extensively in *Discussion section:*

“DBS GPi for dystonias was introduced 2003 in a cohort of patients with generalized or segmental dystonias especially in carriers of DYT1 mutations, in whom treatment options have been limited. Golden responders to DBS GPi were mostly patients carrying a DYT1 mutation. Conversely, short term and long-term DBS effects in carriers of mutations in recently discovered dystonia genes that underwent GPi DBS for generalized or segmental dystonia in young age are seemingly highly variable. Furthermore, the value of pre-interventional genetic testing is currently unclear and DBS target structures for genetically assigned monogenic dystonias are to be enlightened. While the optimal time point of intervention for DBS in dystonias is still under debate, cumulative evidence suggests that an early intervention may decisively contribute to a successful DBS outcome (6-8) in distinct/most monogenic dystonias, raising the issue of a timely genetic diagnosis. “

11. Language: Small errors (randomised or randomized, two or 2). The term “mutant genes” is no longer preferred, but gene variants.

Thank for your appreciation. We have corrected the errors.

1. Rodrigues FB, Duarte GS, Prescott D, Ferreira J, Costa J. Deep brain stimulation for dystonia. Cochrane Database Syst Rev. 2019;1:CD012405.
2. Albanese A, Bhatia K, Bressman SB, DeLong MR, Fahn S, Fung VS, et al. Phenomenology and classification of dystonia: a consensus update. Mov Disord. 2013;28(7):863-73.
3. Petersen JJ, Juul S, Kamp CB, Lokkegaard A, Jakobsen JC. We need to rethink outcomes in Parkinson's disease research. BMJ. 2024;387:q2280.

REFERENCES:

1. Dashtipour K, Mari Z, Jankovic J, Adler CH, Schwartz M, Brin MF. Minimal clinically important change in patients with cervical dystonia: Results from the CD PROBE study. J Neurol Sci. 2019;405:116413.
2. Pintér D, Janszky J, Kovács N. Minimal Clinically Important Differences for Burke-Fahn-Marsden Dystonia Rating Scale and 36-Item Short-Form Health Survey. Mov Disord. 2020;35(7):1218-23.
3. Kupsch A, Benecke R, Müller J, Trottenberg T, Schneider GH, Poewe W, et al. Pallidal deep-brain stimulation in primary generalized or segmental dystonia. N Engl J Med. 2006;355(19):1978-90.
4. Murad MH, Sultan S, Haffar S, Bazerbachi F. Methodological quality and synthesis of case series and case reports. BMJ Evid Based Med. 2018;23(2):60-3.

5. Wells GA SB, O'Connell D, Peterson J, Welch V, Losos M, et al. . The Newcastle-Ottawa Scale (NOS) for assessing the quality of nonrandomised studies in meta-analyses. http://wwwohrica/programs/clinical_epidemiology/oxfordasp Accessed 16 Jan 2020.
6. Artusi CA, Dwivedi A, Romagnolo A, Bortolani S, Marsili L, Imbalzano G, et al. Differential response to pallidal deep brain stimulation among monogenic dystonias: systematic review and meta-analysis. J Neurol Neurosurg Psychiatry. 2020;91(4):426-33.
7. Cheung T, Zhang C, Rudolph J, Alterman RL, Tagliati M. Sustained relief of generalized dystonia despite prolonged interruption of deep brain stimulation. Mov Disord. 2013;28(10):1431-4.
8. Markun LC, Starr PA, Air EL, Marks WJ, Jr., Volz MM, Ostrem JL. Shorter disease duration correlates with improved long-term deep brain stimulation outcomes in young-onset DYT1 dystonia. Neurosurgery. 2012;71(2):325-30.
